# Genetic interactions between ABA signalling and the Arg/N-end rule pathway during Arabidopsis seedling establishment

**DOI:** 10.1038/s41598-018-33630-5

**Published:** 2018-10-12

**Authors:** Hongtao Zhang, Lucy Gannon, Peter D. Jones, Chelsea A. Rundle, Kirsty L. Hassall, Daniel J. Gibbs, Michael J. Holdsworth, Frederica L. Theodoulou

**Affiliations:** 1 Plant Sciences Department, Rothamsted Research, Harpenden, AL5 2JQ UK; 2 School of Biosciences, University of Nottingham, Loughborough, LE12 5RD UK; 3 Present Address: Department of Cardiovascular Sciences, University of Leicester, Leicester, LE3 7QP UK; 4 Computational and Analytical Sciences Department, Rothamsted Research, Harpenden, AL5 2JQ UK; 5 School of Biosciences, University of Birmingham, Edgbaston, B15 2TT UK

## Abstract

The Arg/N-end rule pathway of ubiquitin-mediated proteolysis has multiple functions throughout plant development, notably in the transition from dormant seed to photoautotrophic seedling. PROTEOLYSIS6 (PRT6), an N-recognin E3 ligase of the Arg/N-end rule regulates the degradation of transcription factor substrates belonging to Group VII of the Ethylene Response Factor superfamily (ERFVIIs). It is not known whether ERFVIIs are associated with all known functions of the Arg/N-end rule, and the downstream pathways influenced by ERFVIIs are not fully defined. Here, we examined the relationship between PRT6 function, ERFVIIs and ABA signalling in Arabidopsis seedling establishment. Physiological analysis of seedlings revealed that N-end rule-regulated stabilisation of three of the five ERFVIIs, *RAP2.12*, *RAP2.2* and *RAP2.3*, controls sugar sensitivity of seedling establishment and oil body breakdown following germination. ABA signalling components *ABA INSENSITIVE* (*ABI)4* as well as *ABI3* and *ABI*5 were found to enhance ABA sensitivity of germination and sugar sensitivity of establishment in a background containing stabilised ERFVIIs. However, N-end rule regulation of oil bodies was not dependent on canonical ABA signalling. We propose that the N-end rule serves to control multiple aspects of the seed to seedling transition by regulation of ERFVII activity, involving both ABA-dependent and independent signalling pathways.

## Introduction

**Figure 1:**
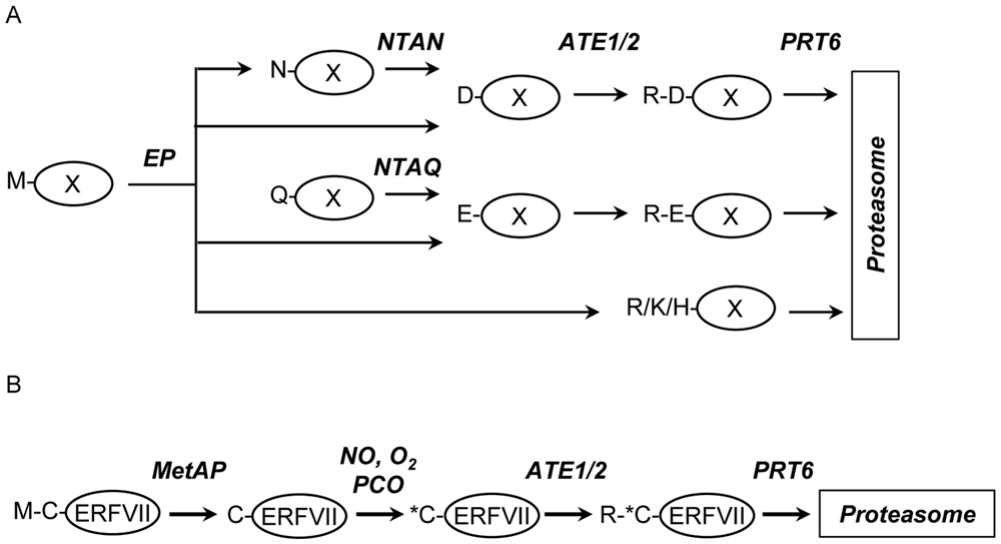
The PRT6 branch of the Arg/N-end rule pathway. (**A**) PRT6 substrates can be generated by the action of endopeptidases (EP). Endopeptidase cleavage may reveal primary destabilising residues (Arg, Lys, His), secondary destabilising residues (Asp, Glu) which are subject to Nt-arginylation or tertiary destabilising residues (Asn, Gln) which are converted to Asp and Glu respectively via specific Nt-amidases (NTAN, NTAQ). Nt arginylation is catalysed by arginyltransferase 1 and 2 (ATE1/2) in Arabidopsis. Single amino acid code is used throughout. (**B**) The Met-Cys initiating ERFVII transcription factors, RAP2.12, RAP2.2, RAP2.3, HRE1, HRE2 (MC-ERFVII) are Arg/N-end rule substrates in Arabidopsis. Met1 is removed by methionine aminopeptidases (MetAP), revealing Nt Cys which is susceptible to oxidation catalysed by plant cysteine oxidases (PCO), a process which also requires nitric oxide (NO). Nt oxidised Cys residues are arginylated by the action of ATE1/2. The resulting basic N-termini are substrates for PRT6 which directs the ERFVII RAP proteins for proteasomal degradation.

The N-end rule pathway relates the fate of a protein to the identity of its amino terminal (Nt) residue. Discovered in 1986, the N-end rule was the first example of targeted protein degradation by the ubiquitin proteasome system (UPS)^1,2^ and has since emerged as an important regulator of diverse processes in eukaryotes^3–6^. The architecture of the pathway is conserved between plant, animal and fungal kingdoms and comprises three branches. The Ac/N-end rule targets proteins containing Nt-acetylated residues, whereas the Pro/N-end rule and the Arg/N-end rule recognise free Nt residues revealed following protein cleavage by non-processive endopeptidases^3,7–9^. Plant genomes encode at least two Arg/N-end rule E3 ligases with different substrate specificities^10^. PROTEOLYSIS1 (PRT1) recognises aromatic Nt residues, Phe, Tyr and Trp, and PROTEOLYSIS6 (PRT6) targets basic Nt residues, Arg, Lys and His^10–13^. Proteins may also become substrates for PRT6 following enzymatic modification of newly-revealed free N-termini. Nt amidases specific for Asn or Gln produce acidic Nt residues, Asp and Glu, respectively, which in turn can be substrates for arginyltransferase (ATE) enzymes that transfer Arg from tRNA^Arg^ to generate basic N-termini (Fig. 1A). Proteins initiating Met-Cys may also be subject to PRT6-mediated degradation following removal of Nt Met by methionine amino peptidases and oxidation of Cys2, catalysed by plant cysteine oxidases^14–16^. The resulting Cys sulfinic acid is then subject to Nt arginylation by ATE^17,18^, thus creating a PRT6 substrate (Fig. 1B).

PRT6 was discovered by homology to the yeast E3 ligase, ScUBR1^10^ and subsequently identified in a genetic screen as a positive regulator of germination in Arabidopsis^19^. Germination of *prt6* mutants is highly hypersensitive to abscisic acid (ABA) and insensitive to the dormancy breaking activity of NO^19,20^. *prt6* seeds and seedlings are hypersensitive to exogenous sugars, contain numerous oil bodies in endosperm and hypocotyls and exhibit dysregulated endosperm storage protein mobilisation^19,21^. Thus, PRT6 is important for several aspects of the transition from seed to seedling, including the mobilisation of seed storage reserves. Characterisation of loss of function alleles at later developmental stages has revealed further roles for the PRT6 branch of the Arg/N-end rule in photomorphogenesis, leaf development, senescence, and responses to abiotic and biotic stress^14,15,20,22–29^. Many of these processes depend on the only known PRT6 substrates, transcription factors belonging to Group VII of the Ethylene Response Factor superfamily (ERFVIIs)^14,15,20,21,25,29^. Arabidopsis has five ERFVII members: *HYPOXIA RESPONSIVE ERF1* (*HRE1*), *HRE2*, *RELATED TO APETALA2.2* (*RAP2.2*), *RAP2.3* and *RAP2.12*, all of which are Met-Cys proteins whose stability is conditional on the oxidation state of Cys2 following co-translational removal of Met1. The turnover of ERFVIIs also requires NO, thus the PRT6 branch of the Arg/N-end rule acts as both an oxygen and an NO sensor^14,15,20^. Stabilised ERFVIIs in *prt6* alleles result in constitutive expression of hypoxia-associated genes and proteins and altered tolerance of hypoxia and submergence^14,15,21,26,30–32^.

A significant challenge for the Arg/N-end rule is to relate known physiological roles to known or novel protein substrates of PRT6 and their downstream signalling pathways. Genetic and physiological studies have shown that the germination phenotype of *prt6* requires *RAP2.12*, *RAP2.2* and *RAP2.3* but not *HRE1* or *HRE2*^20^. The RAP-type ERFVIIs have been proposed to promote seed dormancy and ABA sensitivity by enhancing promoter activity of *ABA INSENSITIVE 5* (*ABI5*) in the endosperm^20^. *ABI5* is a master regulator of ABA signalling, identified together with *ABI3* and *ABI4* in genetic screens for decreased sensitivity to ABA inhibition of germination^33^. *ABI3*, *ABI4* and *ABI5* encode transcription factors belonging to the B3 domain, AP2 domain and bZIP domain classes respectively^34–36^ and act together in a complex regulatory network^37–40^. Whilst originally proposed as seed-specific regulators, diverse roles for *ABI3*, *4* and *5* in vegetative tissue have been demonstrated subsequently^37,41–43^.

In this study, we examined further the relationship between ERFVIIs, ABA signalling and PRT6 action in Arabidopsis seedlings. *ABI4* as well as *ABI3* and *ABI5* was found to contribute to ABA sensitivity of germination and sugar sensitivity of establishment in *prt6*, both of which are ERFVII dependent. Unexpectedly, we found that *ABI4* transcript levels were increased in etiolated *prt6* seedlings in an ERFVII-dependent manner, but *ABI4* did not underpin the ERFVII-dependent delayed greening of *prt6* upon exposure to light. Although the oil body phenotype of *prt6* seedlings was controlled by RAP-type ERFVII transcription factors, it was not dependent on canonical ABA signalling. We propose that the N-end rule serves to control the seed to seedling transition through both ABA-dependent and independent signalling pathways.

## Results

### Ectopic oil bodies in *prt6* are *ERFVII*-dependent but do not require canonical ABA signalling

**Figure 2:**
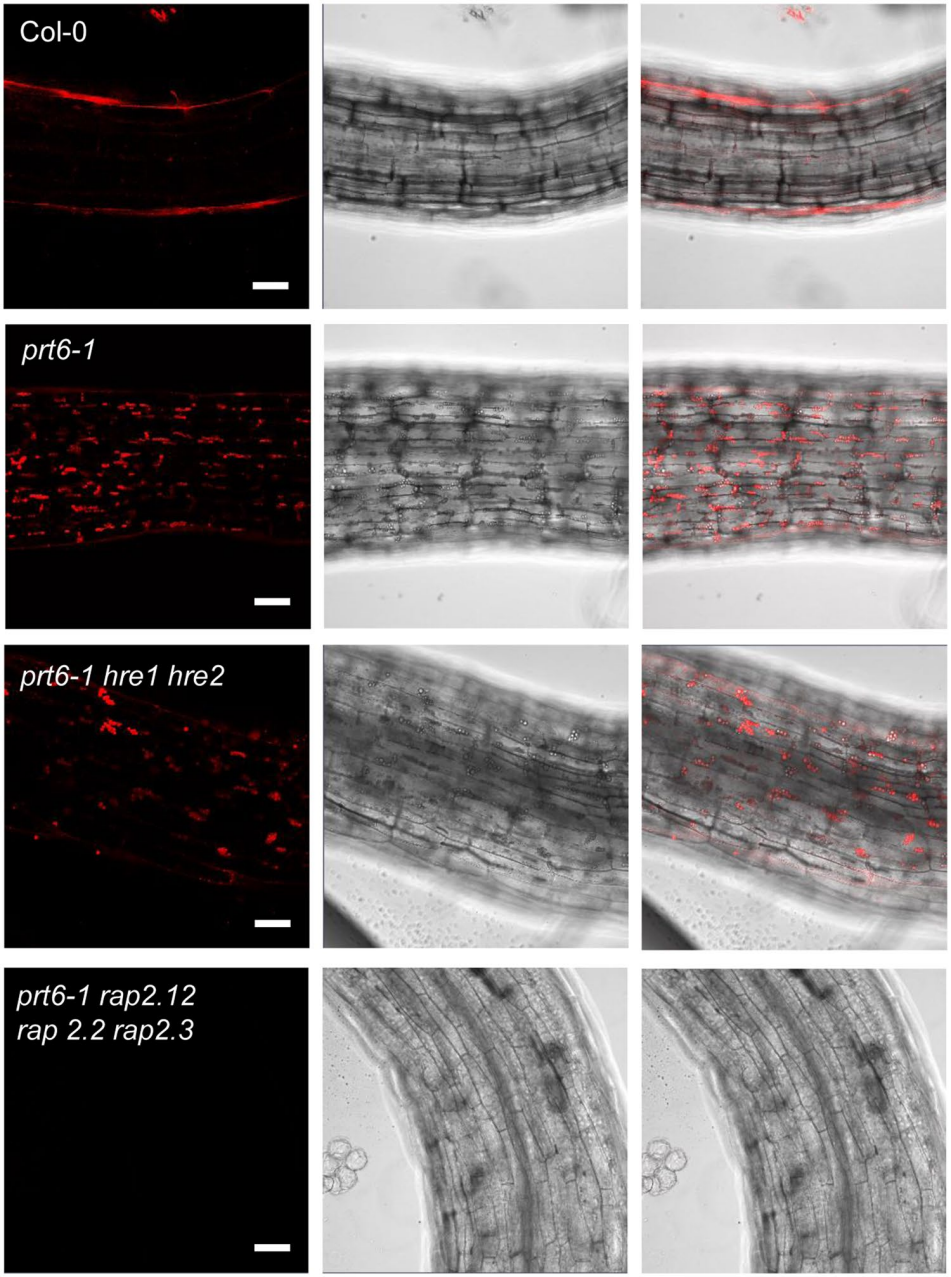
RAP-type ERFVII transcription factors underpin the oil body phenotype of *prt6*. Hypocotyls of 5 d old, light-grown seedlings *prt6* and *erfVII* combination mutants were stained with Nile Red and visualised by confocal microscopy. Left panel: Nile Red; middle panel, bright field, right panel, merge. Bar = 50 μm.

Since the ERFVII transcription factors *RAP2.12*, *RAP2.2* and *RAP2.3* not only control the transition from dormancy to germination^20^, but also play a role in endosperm storage protein mobilisation^21^, we tested whether they underpin the role of the N-end rule in regulating lipid reserve mobilisation. Five days after germination, hypocotyls and endosperm of light-grown *prt6-1* seedlings contained numerous oil bodies, in contrast to Col-0, in which oil bodies were almost completely absent (Fig. 2; Supplementary Fig. S1). The morphology of oil bodies appeared normal in *prt6-1* seedlings (Supplementary Fig. S2). Whereas *prt6-1 hre1 hre2* triple mutant seedlings resembled wild type, removal of *RAP* transcription factor function reverted the *prt6-1* phenotype, indicating that N-end rule pathway-mediated degradation of RAP-type ERFVIIs (but not HRE1 and HRE2) is required for the removal of oil bodies following germination. However, expression of individual stabilised ERFVIIs bearing Ala at residue 2 in place of the destabilising residue Cys, was insufficient to phenocopy *prt6* (Supplementary Fig. S3).

**Figure 3:**
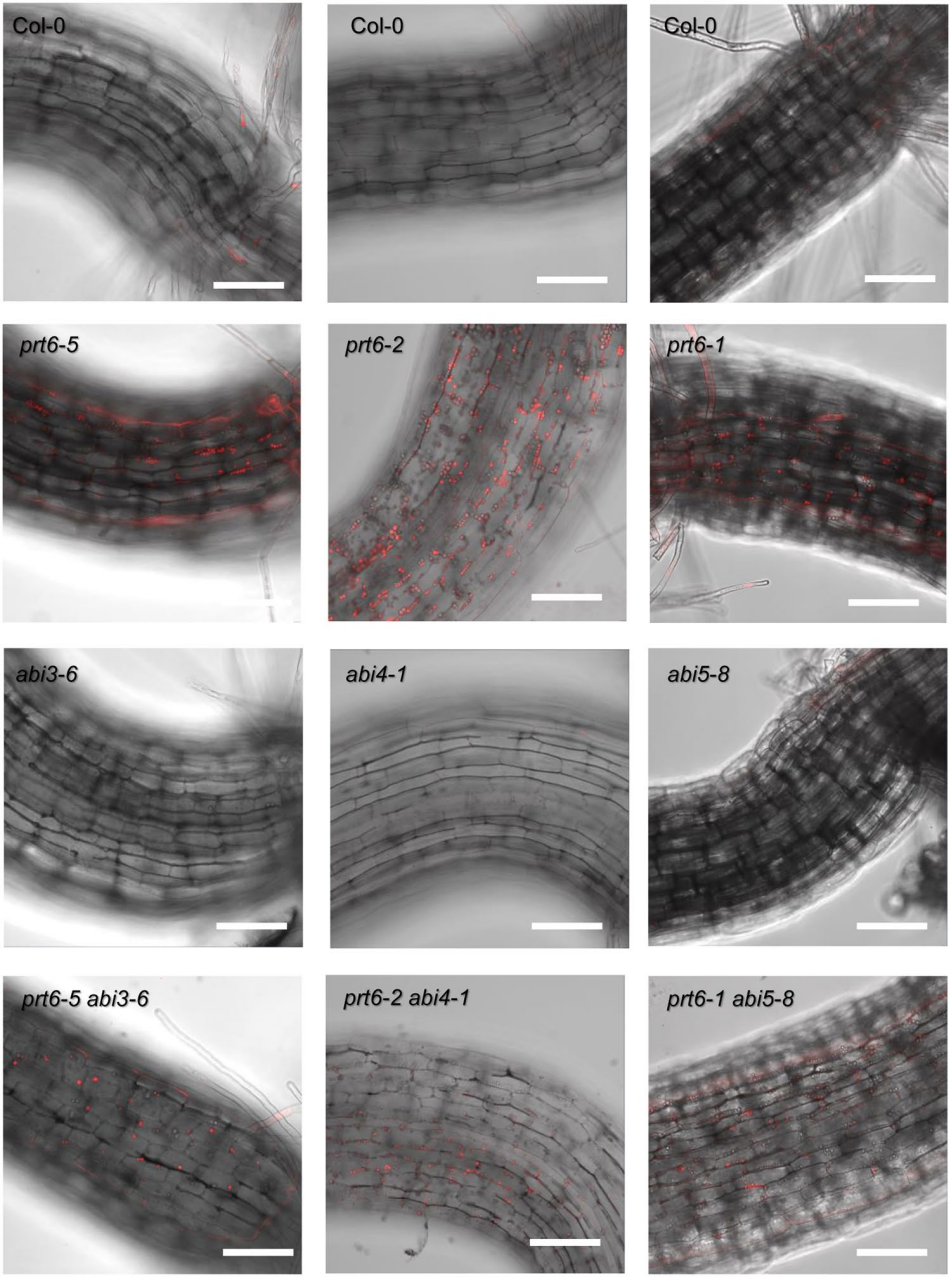
*ABA INSENSITIVE* loci are not required for the oil body phenotype of *prt6*. Hypocotyls of 5 d old, light-grown seedlings were stained with Nile Red and visualised by confocal microscopy. The panels show merged Nile Red and bright field images. Bar = 100 μm.

Previously, we explored the interaction between the N-end rule pathway and ABA signalling via *ABI3* and *ABI5* in the regulation of oil body degradation^19^. As these experiments were conducted with mixed-accession double mutants containing the *prt6–4* allele, a fast-neutron mutant in the Landsberg *erecta* background with a large chromosomal rearrangement, we constructed new double mutant combinations all derived from the Columbia accession. We also constructed a *prt6-2 abi4-1* double mutant to investigate the genetic interactions between *PRT6* and *ABI4*. Details of individual alleles are given in Supplementary Table S1. *prt6-1*, *prt6-2* and *prt6-5* are T-DNA insertion null alleles which are functionally interchangeable^19,21,24^. *prt6-2* seedlings exhibited a similar phenotype to *prt6-1* and *prt6-5* single mutants, with numerous oil bodies in hypocotyl cells (Fig. 3). Oil bodies were largely absent from Col-0 and *abi4-1* seedlings but were retained in the *prt6-2 abi4-1* double mutant, indicating that *ABI4* does not play a role in N-end rule regulated oil body degradation. Similarly, both *prt6-5 abi3–6* and *prt6-1 abi5–8* double mutants contained oil bodies (Fig. 3). As *abi5–8* is not a complete loss of function allele, we also crossed *prt6–1* to the stronger allele, *abi5-1*^36^, which was used in our earlier study^19^. Numerous oil bodies were also present in *prt6–1 abi5–1* hypocotyls, consistent with the notion that *ABI5* function is not required for the *prt6* oil body phenotype (Supplementary Fig. S4). Three SnRK kinases, Snrk2.2, 2.3 and 2.6 are essential for ABA signalling in germination^44,45^ and form part of the core ABA signalling pathway^46^. Whilst hypocotyls of the triple *snrk2.2 2.3. 2.6* mutant lacked oil bodies, removal of *SnRK*2 function in the *prt6–1* background did not revert the wild type phenotype (Supplementary Fig. S5), indicating that the presence of oil bodies in *prt6* mutants does not require canonical ABA signalling.

### ERFVII transcription factors interact with ABA signalling to control sucrose sensitivity of seedling establishment

**Figure 4:**
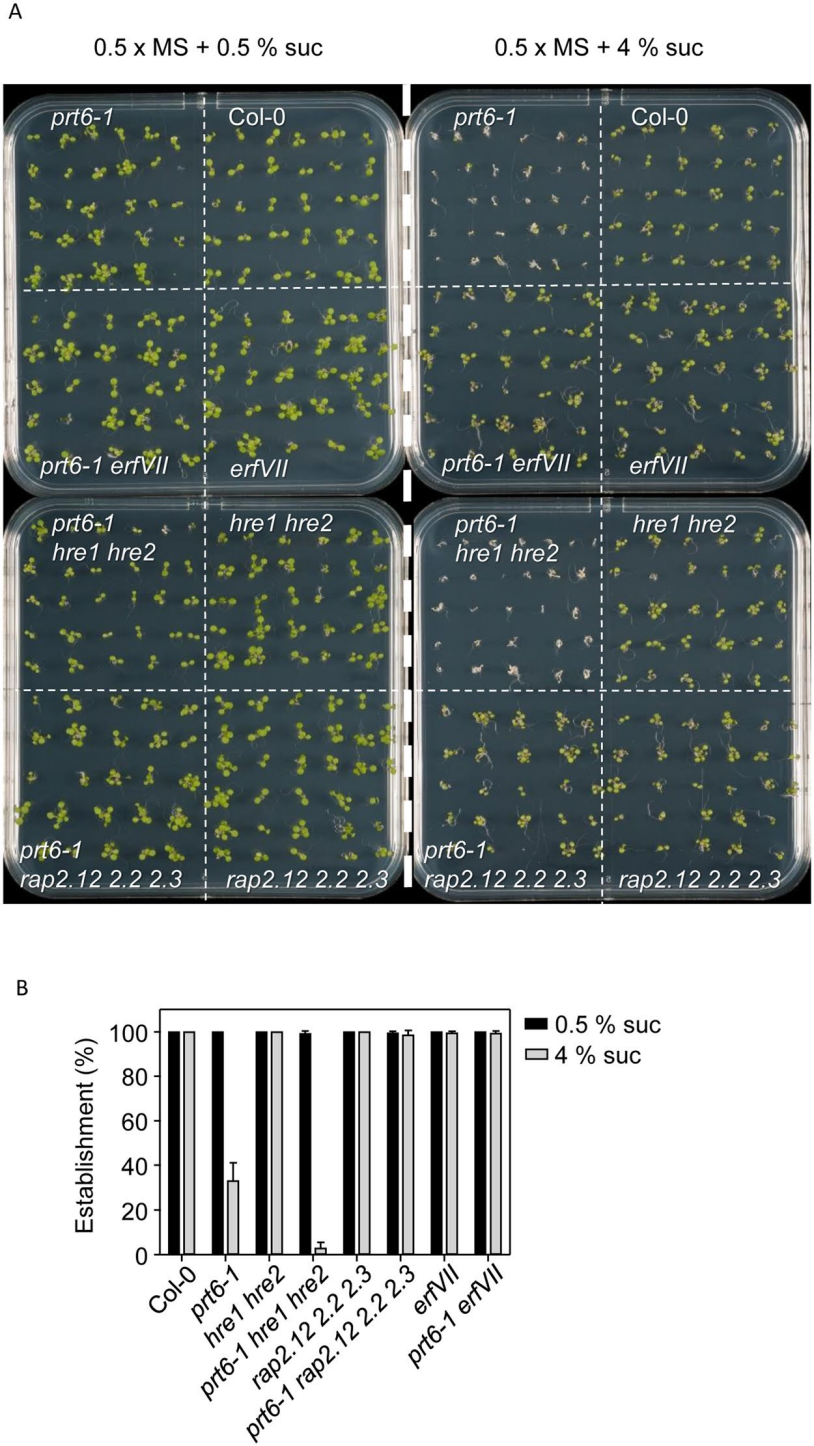
Sugar sensitivity of *prt6* seedling establishment requires ERVII transcription factors. Seeds of *prt6* and *erfVII* combination mutants were germinated on 0.5 x MS medium containing 0.5% or 4% sucrose. (**A**). Images of seedlings grown for 5 d in long days on 0.5 x MS medium containing 0.5% or 4% sucrose. (**B**). Seedling establishment was scored after for 5 d growth under long days. Values are means ± SD (n = 3). No significant differences were observed in establishment on 0.5% sucrose (one-way Kruskal-Wallis ANOVA; P = 0.51, Χ^2^, 7 df); differences were observed on 4% sucrose (one-way Kruskal-Wallis ANOVA, p = 0.01, Χ^2^, 7 df), with establishment of *prt6* and *prt6 hre1 hre2* consistently lower than other genotypes.

**Figure 5:**
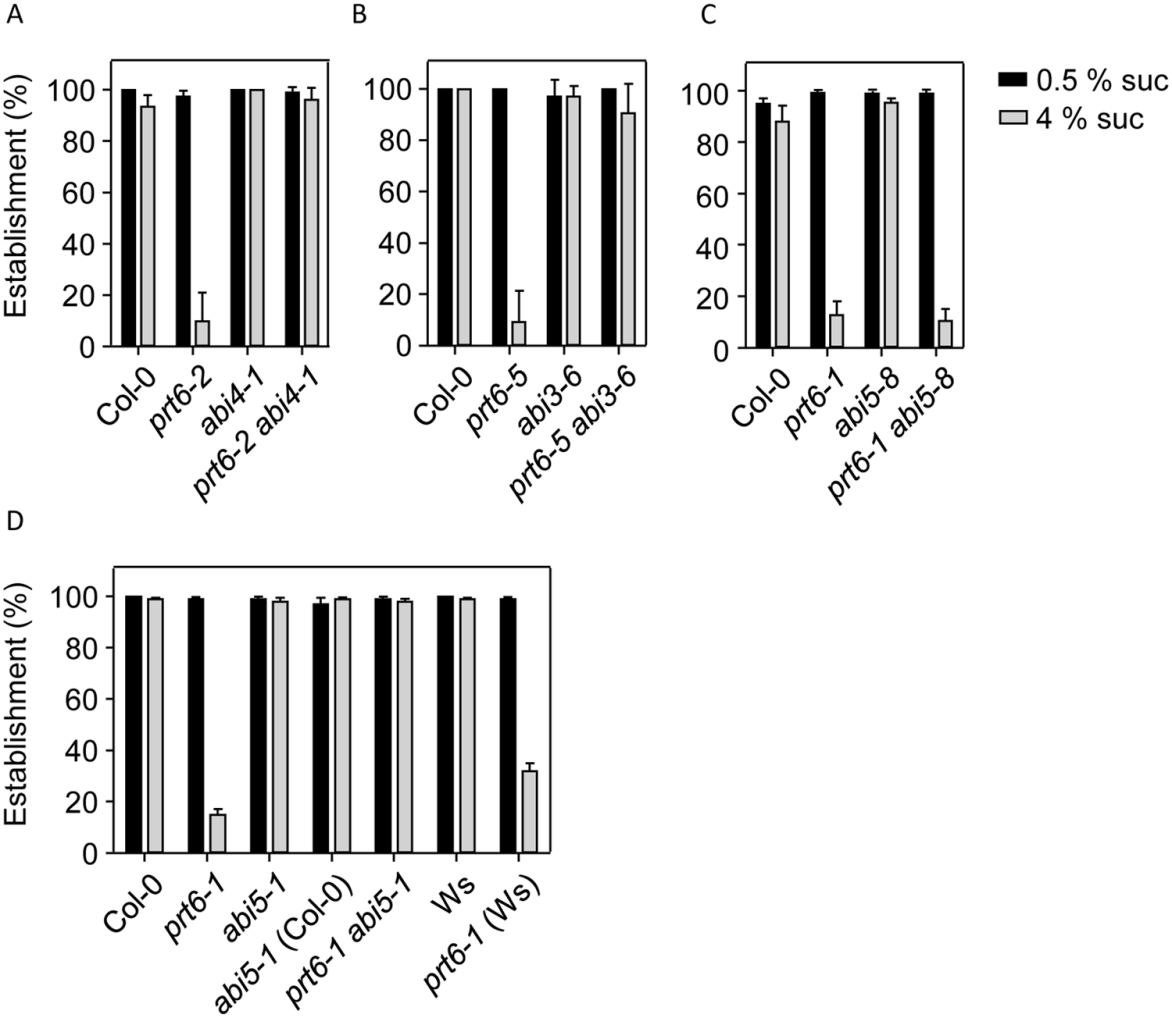
Sugar sensitivity of *prt6* seedling establishment requires ABA signalling. Seeds of *prt6* and ABA signalling combination mutants were germinated on 0.5 x MS medium containing 0.5% or 4% sucrose. Seedling establishment was scored after for 5 d growth under long days. Values are means ± SD (n ≥ 3). No significant differences were observed in establishment on 0.5% sucrose, according to one-way Kruskal-Wallis ANOVA; differences were observed on 4% sucrose (A, p = 0.0179, 3 df; B, p = 0.0228, 3 df; C, p = 0.0345, 3 df; D, p = 0.0347, 6 df).

Several genetic studies have indicated a role for ABA in sugar signalling during seedling establishment^47–50^ and our previous study indicated opposite genetic functions for *PRT6* and both *ABI3* and *ABI5* in this response^19^. Therefore, we analysed genetic interactions between *PRT6* and *ABI4* for sugar sensitivity of seedling establishment. We also tested whether the sugar hypersensitivity of *prt6* was *ERFVII* dependent. Wild type seedlings exhibited 100% establishment on 0.5 X MS plates containing 4% sucrose but establishment of *prt6–1* seedlings was severely impaired on this medium (Fig. 4). The triple *prt6–1 hre1 hre2* mutant also failed to establish on high sucrose medium but *prt6-1 rap2.12 rap2.2 rap2.3* seedlings resembled wild type, indicating that RAP-type *ERFVIIs* are required for sucrose sensitivity. Seedlings of all genotypes established on 0.5% sucrose (Fig. 4). All null *prt6* alleles, *prt6–1*, *prt6–2* and *prt6–5* were sensitive to sucrose for seedling establishment (Fig. 5; Supplementary Fig. S6). However, establishment of *prt6-2 abi4-1* and *prt6-5 abi3–6* double mutants was not significantly different to wild type (Fig. 5A,B; Supplementary Fig. S6). The *prt6-1 abi5–8* mutant exhibited similar sucrose sensitivity to *prt6-1* (Fig. 5C; Supplementary Fig. S6) but combining *prt6-1* with a stronger *abi5* allele, *abi5-1* resulted in 98% establishment on high sugar (Fig. 5D; Supplementary Fig. S7). Therefore, *ABI3*, *ABI4* and *ABI5* are all required for sucrose hypersensitivity of *prt6*.

**Figure 6:**
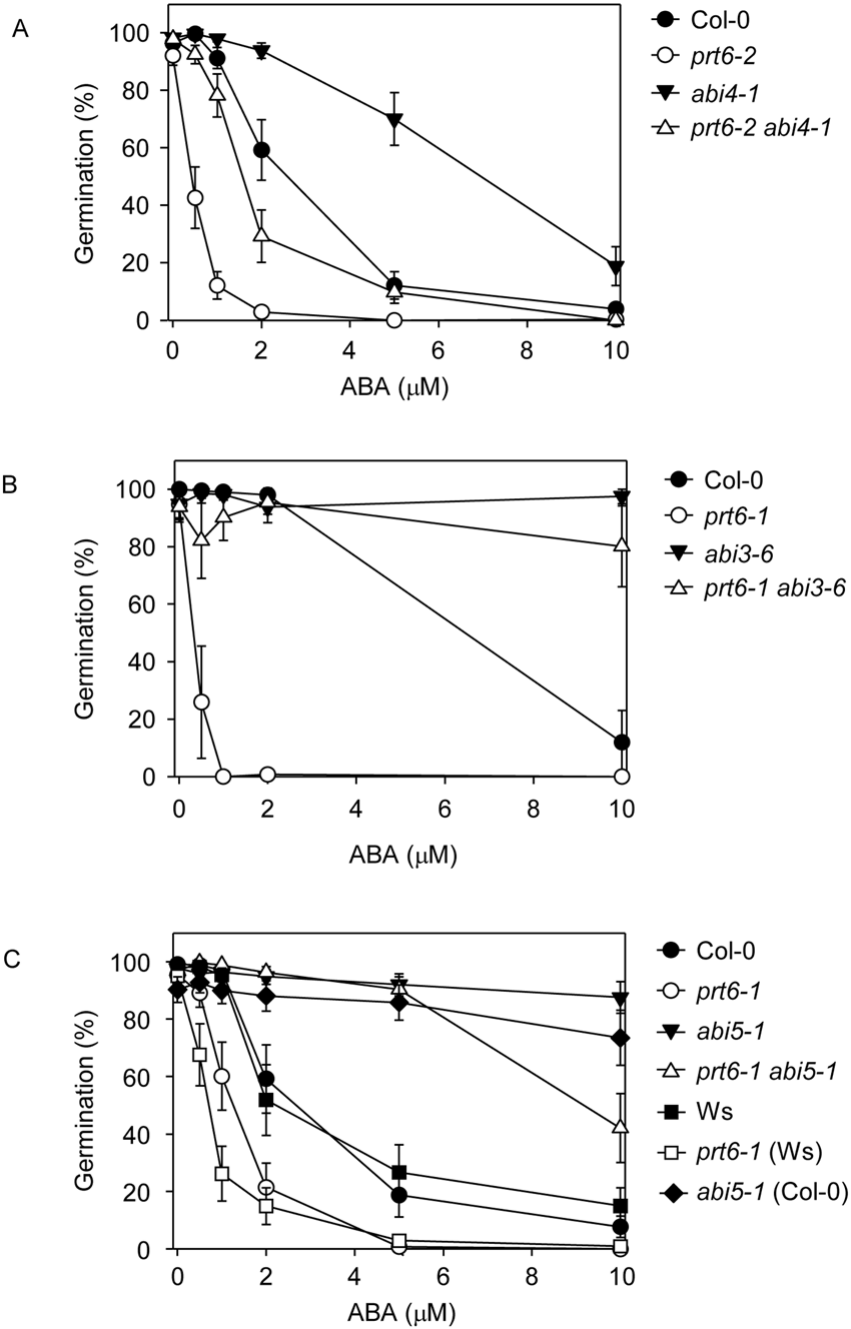
*ABA INSENSITIVE* loci contribute to the ABA hypersensitivity of *prt6* seed germination. Seeds of the indicated genotypes were plated on 0.5 X MS containing the indicated amounts of ABA and germination scored after 7 d in continuous light. Values are predicted mean germination rates with approximate standard errors (n ≥ 3). Error bars are calculated as the approximate back-transformation of the estimated standard errors obtained from the linear mixed model fitted to the logit germination rate. Note that the ABA sensitivity of *prt6* alleles declines with seed age^19^. Fresh seeds collected from yellow siliques were used for the experiment shown in panel B, since the *abi3–6* mutation negatively impacts seed longevity. Seeds for the experiments shown in A and C were stored for 50–60 weeks.

We also explored a role for *ABI4* in the germination response to ABA. *abi4-1* seeds exhibited 70% germination in the presence of 5 μM ABA, a concentration that strongly inhibited germination of Col-0. Consistent with previous data, *prt6-2* seeds were hypersensitive to ABA (Fig. 6A). This sensitivity was partially relieved in the *prt6-2 abi4-1* double mutant, which showed 29% germination on 2 μM ABA and was still able to germinate on 5 μM ABA (Fig. 6A). As shown previously^19,20^, loss of *ABI3* and *ABI5* function reverts the ABA hypersensitivity of *prt6* germination (Fig. 6B,C).

### *ABI4* is ectopically expressed in etiolated *prt6* seedlings

**Figure 7:**
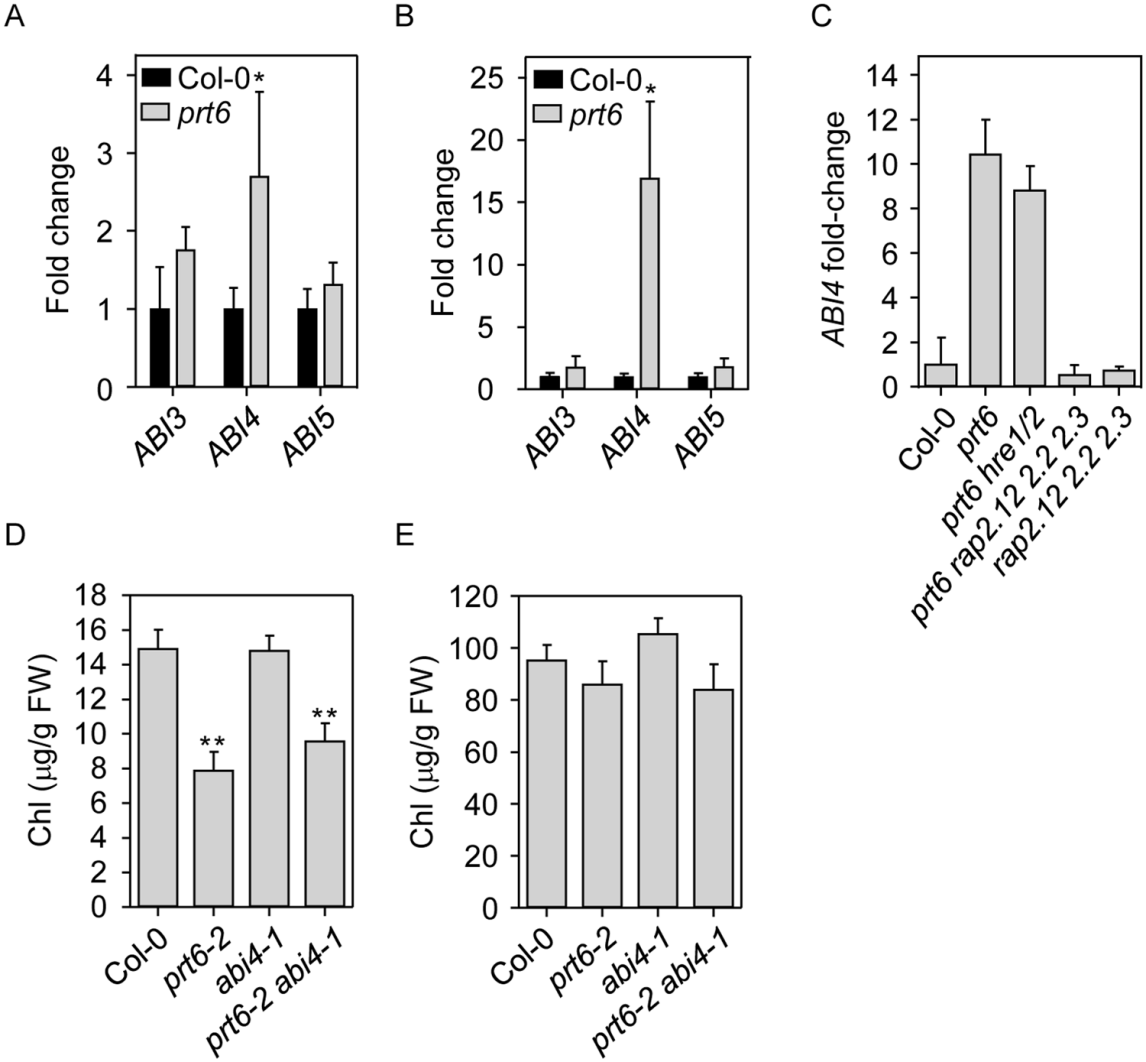
*ABI4* is upregulated in etiolated *prt6* seedlings but does not underpin the delayed regreening phenotype. (**A–C**) Relative transcript abundance determined by quantitative RT-PCR in (**A**) 4 d-old, light grown seedlings and (**B,C**) 4 d-old etiolated seedlings of N-end rule and *ERFVII* mutants. A, B. Expression of *ABI3*, *ABI4*, *ABI5*. Values are means ± SD (n = 4); * indicates p < 0.05, Mann-Whitney U test. (**C**) Expression of *ABI4*. Values are means ± SD (n = 3); p < 0.05, one-way Kruskal-Wallis ANOVA. (**D,E**) Total chlorophyll in seedlings 5 h (**D**) and 24 h (**E**) after transfer to light. Values are means ± SE (n = 4). ** indicates p < 0.01 from the ANOVA contrast with 1 and 10 df.

Overexpression of *ABI3*, *ABI4* and *ABI5* is known to confer hypersensitivity to ABA and sugar^37,42,51^ and *ABI5* promoter::GUS activity was enhanced specifically in the endosperm of after-ripened *prt6* seeds^20^. Therefore, we used quantitative reverse-transcriptase PCR to test whether expression of *ABI3*, *4* and 5 was enhanced in *prt6* seedlings and whether this was associated with sugar sensitivity or other *prt6* phenotypes. In light-grown seedlings, expression levels of the *ABI3* and *ABI5* transcripts were not significantly different between *prt6* and WT, but significant differences were observed for *ABI4* which showed a modest increase in *prt6* (Fig. 7A). In contrast, the observed differences in *ABI4* expression in etiolated *prt6* seedlings showed a marked increase in *prt6* with no differences observed in *ABI3* and *ABI5* transcript levels (Fig. 7B). The observed differences in *ABI4* expression in etiolated tissue showed an increase dependent on *RAP*-type *ERFVIIs* but not *HRE1* and *HRE2* (Fig. 7C).

To explore the physiological significance of this upregulation, we investigated a potential interaction between *ABI4* and *PRT6* in de-etiolation, since they play opposing roles in this process. *ABI4* stimulates hypocotyl elongation in the dark and represses photosynthetic gene expression^52,53^ whereas *PRT6* promotes photomorphogenesis by relieving *ERFVII*-mediated repression of chlorophyll biosynthetic genes and proteins^15,21^. Thus, *prt6* seedlings green more slowly upon illumination in a *ERFVII RAP*-dependent manner (Supplementary Fig. S8)^25^. Removal of *ABI4* function in the *prt6* background did not rescue the slow greening phenotype, as judged by chlorophyll measurements (Fig. 7D), however there was a genetic interaction between *prt6-2* and *abi4-1*, resulting in a wavy seedling phenotype (Supplementary Fig. S8). After 24 h light all genotypes had comparable levels of chlorophyll (Fig. 7E).

## Discussion

The PRT6 branch of the N-end rule pathway has multiple functions throughout plant development, particularly in the transition from dormant seed to photoautotrophic seedling. RAP-type ERFVII transcription factors control the germination phenotype of *prt6* seeds^20^ and play a role in regulating breakdown of endosperm storage protein reserves^21^. *prt6* endosperm and hypocotyls contain oil bodies at a developmental stage when they have been degraded in WT^19^ (Fig. 2). In the current study, we demonstrate that the oil body phenotype of *prt6* seedlings is also *ERFVII*-dependent. *RAP2.12*, *2.2* and *2.3* were required for the presence of oil bodies in *prt6* endosperm and hypocotyls, but expressing individual stabilised ERFVIIs did not lead to the appearance of ectopic oil bodies in wild type seedlings (Supplementary Fig. S3), indicating that stabilisation of more than one transcription factor might be required to recapitulate the oil body phenotype.

*ABI5* has been identified as an ERFVII RAP target via chromatin immunoprecipitation and transactivation assays^20^, and previously we published evidence for the association of *ABI3* and *ABI5* with the oil body phenotype of *prt6-4*^19^. However, upon re-examination using double mutants in the same accession background, no evidence for a role for *ABI3* or *ABI5* was found. Although *prt6* hypocotyls contained oil bodies in the absence of *ABI3* function (Fig. 3), the oil bodies in *prt6-1 abi3-6* hypocotyls were fewer in number than those in *prt6-1*, consistent with the fact that *ABI3* promotes expression of oleosins, and *abi3* mutant seeds have reduced levels of storage protein and triacylglycerol^33,54,55^.

In Arabidopsis, endosperm reserve mobilisation is independent of ABA, whereas embryo lipid mobilisation is regulated by *ABI4*^56,57^. *ABI4* not only acts as a repressor of embryo lipid mobilisation but also plays a role in inducing ectopic triacylglycerol biosynthesis under nitrogen stress, through activation of *DGAT1* which encodes the rate-limiting enzyme, diacylglycerol O-acyltransferase 1,^57,58^. However, neither *ABI4* nor the three major ABA-associated SnRK kinases in germinating seeds were required for presence of oil bodies in *prt6* seedlings (Fig. 3, Supplementary Fig. S5). Taken together, these results indicate that the ERFVII transcription factors do not regulate oil breakdown via ABA signalling. The *prt6* oil body phenotype may reflect delayed lipid catabolism, increased lipid synthesis or a combination of both processes. *prt6* seedlings are sensitive to 2,4-dichlorophenoxybutyric acid, a synthetic auxin that is converted by β-oxidation to the herbicide 2,4-dichlorophenoxyacetic acid, suggesting that the fatty acid breakdown is not markedly impaired^19^. Neither lipid catabolic nor biosynthetic genes exhibit differential regulation in published transcriptome data from *prt6* seeds and seedlings^14,23^, but a microarray analysis of *ged1*, a *prt6* loss of function mutant in the Ws background revealed that transcripts representing seven of the eight Arabidopsis oleosin genes are down-regulated in *ged1* seedlings (dark-grown for 5 d and then returned to the light for 2 d)^23^. As oleosin is the major structural protein of oil bodies, one would predict that *ged1* would not accumulate oil bodies. However, under our growth conditions, *ged1* hypocotyls contain large numbers of oil bodies, comparable to the *prt6* alleles reported in our manuscript (Supplementary Fig. S9). Moreover, in a previous study, we detected increased levels of Oleosin 1 protein in 4 d old etiolated *prt6* seedlings by immunoblotting^21^. Thus, the phenotype of the *ged1* mutant reported in^23^ is different to that of *ged1* and other null *prt6* alleles grown under our conditions, and thus the ERVII RAP targets that underpin the oil body phenotype remain to be discovered.

Previously, we demonstrated that *prt6* seeds are highly hypersensitive to ABA for germination completion although not for inhibition of root elongation^19^. Germination assays using different alleles of *prt6* confirmed our previous observations^19,20^ that *ABI5* and *ABI3* are both required for ABA sensitivity of *prt6* germination and additionally revealed that *ABI4* also contributes to ABA sensitivity of *prt6* seeds (Fig. 6). All three transcription factors contributed to the sugar sensitivity of *prt6* seedling establishment, which was also dependent on *RAP*-type *ERFVII*s (Figs 4, 5). Although overexpression of *ABI3*, *ABI4* or *ABI5* can produce sugar sensitive phenotypes in seeds and vegetative tissues^37,42,51^, these genes were not markedly over expressed in light grown seedlings (Fig. 7).

Unexpectedly, we found that *ABI4* transcript levels were increased in etiolated seedlings of *prt6*, in an *ERFVII RAP*-dependent manner (Fig. 7). Although both *ABI4* and *RAP*-type *ERFVII*s repress photosynthetic genes^25,52^, apparently the *ERFVII* transcription factors that are stabilised in *prt6* seedlings repress photomorphogenesis independently of *ABI4*, since the *prt6-2 abi4-1* double mutant displays a slow regreening phenotype (Fig. 7). Thus, the physiological significance of *ABI4* over-expression in etiolated *prt6* seedlings is not clear. *prt6* seedlings do not phenocopy *ABI4* over-expressing transgenic lines: for example, of the 45 protein groups up-regulated in etiolated *prt6* seedlings^21^, only five are encoded by known targets of *ABI4* (^39^, 2011; Supplementary Table S2). In part, this may reflect tight post-translational regulation of *ABI4*, as reported by Finkelstein *et al*.^59^, who demonstrated that *ABI4* transcript and protein levels are poorly correlated. Whilst *ABI4* expression is promoted in the dark by *GUN1* and *PTM*^60^, the E3 ligase COP1 mediates proteasomal degradation of ABI4 protein in the light, enabling activation of photosynthetic genes^53^. In this way, COP1 may override any effect of *ABI4* over-expression in *prt6* seedlings upon transfer to light.

**Figure 8:**
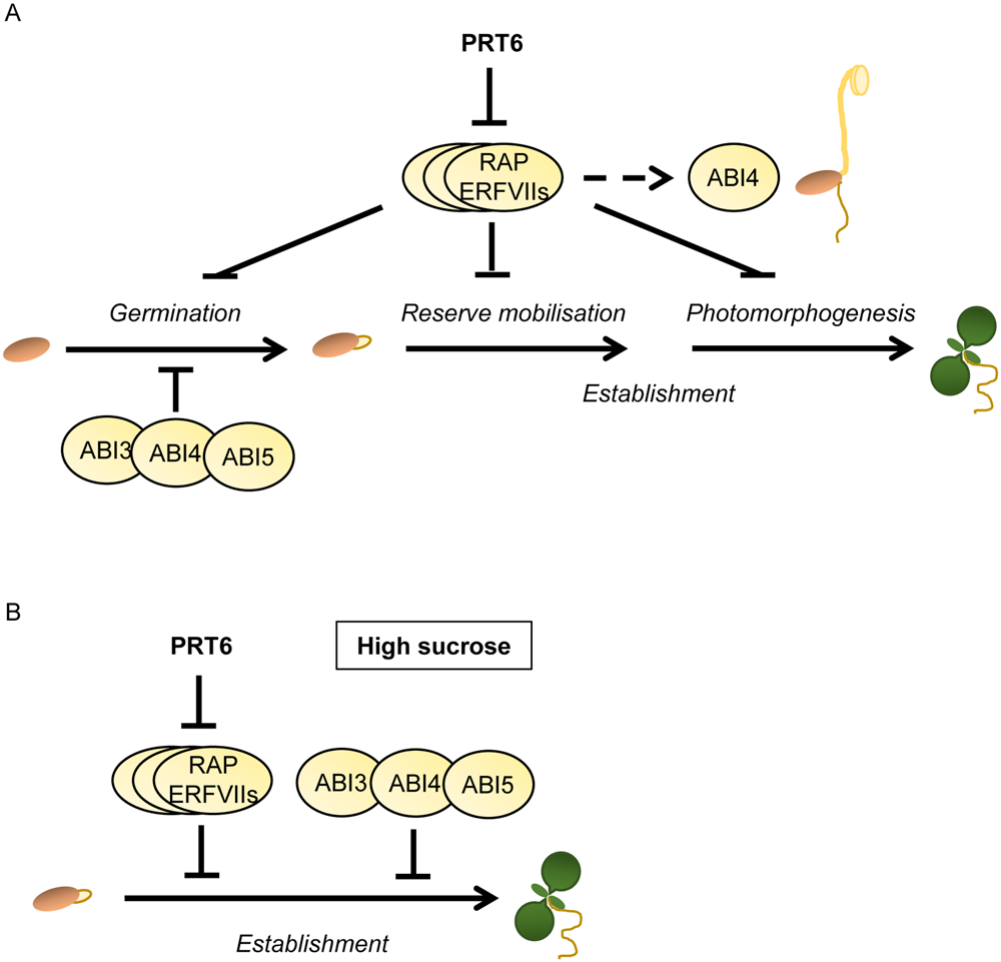
PRT6 regulation of germination and seedling establishment. Model depicting regulation by *PRT6* of different processes identified or confirmed in this study. (**A**) In wild type plants, PRT6 promotes germination by mediating proteasomal degradation of ERFVII transcription factors, which repress germination when stabilised. Germination of *prt6* seeds is hypersensitive to inhibition by ABA, a phenotype that requires the action of ABI3, ABI4 and ABI5 transcription factors. PRT6 and ERFVIIs play several roles in seedling establishment: ERFVII stabilisation in the absence of *PRT6* function leads to an accumulation of oil bodies in endosperm and hypocotyls, indicative of a role for PRT6 in reserve mobilisation. Previously, ERFVIIs have been shown to repress photomorphogenesis in *prt6* seedlings, which have a slow greening phenotype. Neither of the establishment functions of PRT6 appears to involve *ABA INSENSITIVE* (*ABI*) loci, although *ABI4* is known to repress photosynthetic gene expression and the current study demonstrated that *ABI4* transcripts are up-regulated in etiolated *prt6* seedlings, in an *ERFVII*-dependent manner. (**B**) Establishment of *prt6* seedlings is hypersensitive to exogenous sucrose, an effect that requires *ERFVII* and *ABI* action.

We have shown for the first time that the established PRT6 substrates, RAP2.12, RAP2.2 and RAP2.3 are required for two different functions of the Arg/N-end rule pathway in the seed to seedling transition: regulation of oil bodies and sensitivity to exogenous sugar (summarised in Fig. 8). A genetic interaction between the N-end rule pathway and *ABI4* was also identified in the regulation of seedling ABA and sugar sensitivity. Whilst the PRT6 branch of the Arg/N-end rule interacts with ABA signalling during germination and seedling establishment, canonical ABA signalling was not important for the retention of oil bodies in a high ERFVII environment, pointing to diverse roles of the ERFVII transcription factors downstream of the Arg/N-end rule pathway. The identification of novel ERFVII targets associated with different functions of PRT6 will be an important topic for future investigation.

## Materials and Methods

### Arg/N-end rule mutant alleles and transgenic lines

Details of mutant alleles are given in Supplementary Table S1. *prt6-1*, *prt6-2 and prt6-5* mutants are null T-DNA alleles described in^19,23^, and^24^. *ged1* is an EMS allele described in^26^. Combinations of Group VII *ERF* alleles, *prt6-1* and *abi5-8* are described in^14,20^, and^25^. The *prt6-1 snrk2.2 2.3 2.6* is described in^29^. Transgenic lines expressing MA-HRE1, MA-HRE2 and MA-RAP2.3 in Col-0 are described in^14^ and^20^. To generate MA-RAP2.12 and MA-RAP2.2 lines driven by the *35SCaMV* promoter, full-length cDNAs were amplified from *Arabidopsis* total seedling cDNA, ligated into the Entry vector pE2c, and then transferred into the Destination binary vector pB2GW7^61^. The N-terminal mutation was incorporated using the forward primer (Table S3). Transformation into *Agrobacterium tumefaciens* (strain GV3101 pMP90) and *Arabidopsis thaliana* was performed according to established protocols, and transgenic plants were taken through to T_3_ homozygous stage using BASTA selection.

### Construction and validation of double mutants

Double mutants were generated by genetic crosses and *prt6-1*, *prt6-2* and *prt6-5* genotypes were confirmed by T-DNA insertion-based PCR as in^24^. *ged1* was genotyped using primers in Table S3. *abi3-6* was crossed to both *prt6-5* and *prt6-1*; double mutants were maintained in the heterozygous state as *prt6 abi3-6* +/− until required. The *abi3–6* mutation was confirmed by PCR using primer pair abi3–6F3 + abi3–6R6 as in^62^. The *abi4-1* and *abi5-1* mutations were confirmed by CAPS makers: for *abi4-1*, the PCR product of abi4-1F1 and abi4-1R1 was restricted with *Bsp* LI and for *abi5-1*, the product of abi5-1LPavaII and abi5-1RPavaII was restricted with *Ava* II. Controls for experiments employing the mixed accession double mutant, *prt6-1 abi5-1* were obtained by segregation.

### Visualisation of oil bodies

Seedlings were grown and treated as described in^63^. Briefly, seeds were sterilised and plated on 0.5 x MS medium. After incubation for 2–3 d at 4 °C in the dark, plates were transferred to a long day (16 h/8 h) cabinet at 22 °C for 5 d. Seedlings were stained with Nile Red (1 µg/ml) for 1 min, followed by washing with distilled water and placed on microscope slides, and sealing with a cover slip. Oil bodies were imaged using a Zeiss LSM 780 confocal microscope, excitation laser 514 nm; emissions collected between 597–650 nm. Data presented are representative of several independent experiments.

### Germination and seedling establishment assays

Seeds were raised from plants grown in long day conditions (16 h/8 h at 22 °C) as described in;^21^ all genotypes to be compared were raised in the same cabinet. ABA sensitivity of germination was determined using 0.5 X MS medium containing 0–10 μM ABA. Seeds were sterilised as described in^63^ and stratified for 2 d at 4 °C before transfer to 22 °C under continuous light. Germination is defined as emergence of the radicle (embryonic root) from the seed coat. A linear mixed model was fitted using REML to the logit transformation of percentage germination, using an offset of 0.5. A minimum of three seed batches (50 seeds per batch, except for *prt6 abi3–6*; 25 seeds per batch) was used for each line, with the same batch used for all ABA concentrations. Thus, seed batch was included as a random term. Fixed effects were assessed using approximate F-statistics^64^ and included the 2 × 2 factorial structure to assess whether the *abi* and *prt6* mutations acted independently across the ABA concentrations. Sugar sensitivity was assessed by germinating seeds as above on 0.5 X MS medium containing 0.5% or 4% sucrose. Following 5 d growth in long day conditions, establishment was scored as the development of photosynthetic competence (green cotyledons). Experiments were carried out in triplicate, using a minimum of 25 seeds per replicate. A non-parametric one-way Kruskal-Wallis ANOVA was applied to the set of different genotypes for each sugar condition. The associated test statistics (calculated after an adjustment for ties) were evaluated against a chi-squared distribution.

### Real time quantitative reverse-transcription PCR (RT-q-PCR)

RNA was extracted from 4 d old etiolated or light grown seedlings using an RNeasy Plant Mini Kit (Qiagen) and treated with RQ1 RNase-free DNase (Promega). Transcriptor First Strand cDNA Synthesis Kit (Roche) and anchored -oligo(dT)_18_ were used for cDNA synthesis for a two-step RT-PCR. Real-time PCR was conducted with Faststart Essential DNA Green Master mix (Roche) using Lightcycler®96. The relative quantification was done using reference genes *ACT2* (At3g18780.2) and *TUB4* (At5g44340.1) as described in^21^. Gene expression data were normalised by the average expression levels of Col-0 and either a non-parametric Mann-Whitney U-test or a non-parametric Kruskal-Wallis ANOVA was applied to the data. Primers used are given in Supplementary Table S3.

### Chlorophyll determination

Seedlings were immersed in 1.5 ml of 80% Acetone for 24 h at 4 °C in darkness. The extract was subjected to spectrophotometric measurements at 663 and 645 nm using a 6715 UV/VIS Spectrometer (Jenway). Total chlorophyll was calculated using the equation: (20.2x A_645_) + (8.02x A_663_)^65^ and standardized to the fresh weight of seedling tissue (µg of chlorophyll per g of seedling fresh weight). Data were analysed by ANOVA, with orthogonal contrasts incorporated to extract the specific comparisons of interest.

## Electronic supplementary material

Supplementary information

## Data Availability

All data generated or analysed during this study are included in this published article (and its Supplementary Information files).
