# Supplementary material for: Genetic interactions between ABA signalling and the Arg/N-end rule pathway during Arabidopsis seedling establishment
Source: Sci Rep. 2018 Oct 12;8:15192. doi: 10.1038/s41598-018-33630-5 (PMC6185960; doi:10.1038/s41598-018-33630-5)
Supplement: Supplementary file 1 — Supplementary information [file 41598_2018_33630_MOESM1_ESM.pdf]

Supplementary Information

Genetic interactions between ABA signalling and the Arg/N-end rule pathway during Arabidopsis seedling establishment.

Hongtao Zhang[1], Lucy Gannon[1], Peter D. Jones[2,3], Chelsea A. Rundle[1], Kirsty L. Hassall[4], Daniel J. Gibbs[5], Michael J. Holdsworth[2], Frederica L. Theodoulou[1,*]

1. Plant Sciences Department, Rothamsted Research, Harpenden, AL5 2JQ, UK
2. School of Biosciences, University of Nottingham, Loughborough, LE12 5RD, UK
3. Current address: Department of Cardiovascular Sciences, University of Leicester, LE3 7QP, UK
4. Computational and Analytical Sciences Department, Rothamsted Research, Harpenden, AL5 2JQ, UK
5. School of Biosciences, University of Birmingham, Edgbaston, B15 2TT UK

* Corresponding author. Email: freddie.theodoulou@rothamsted.ac.uk

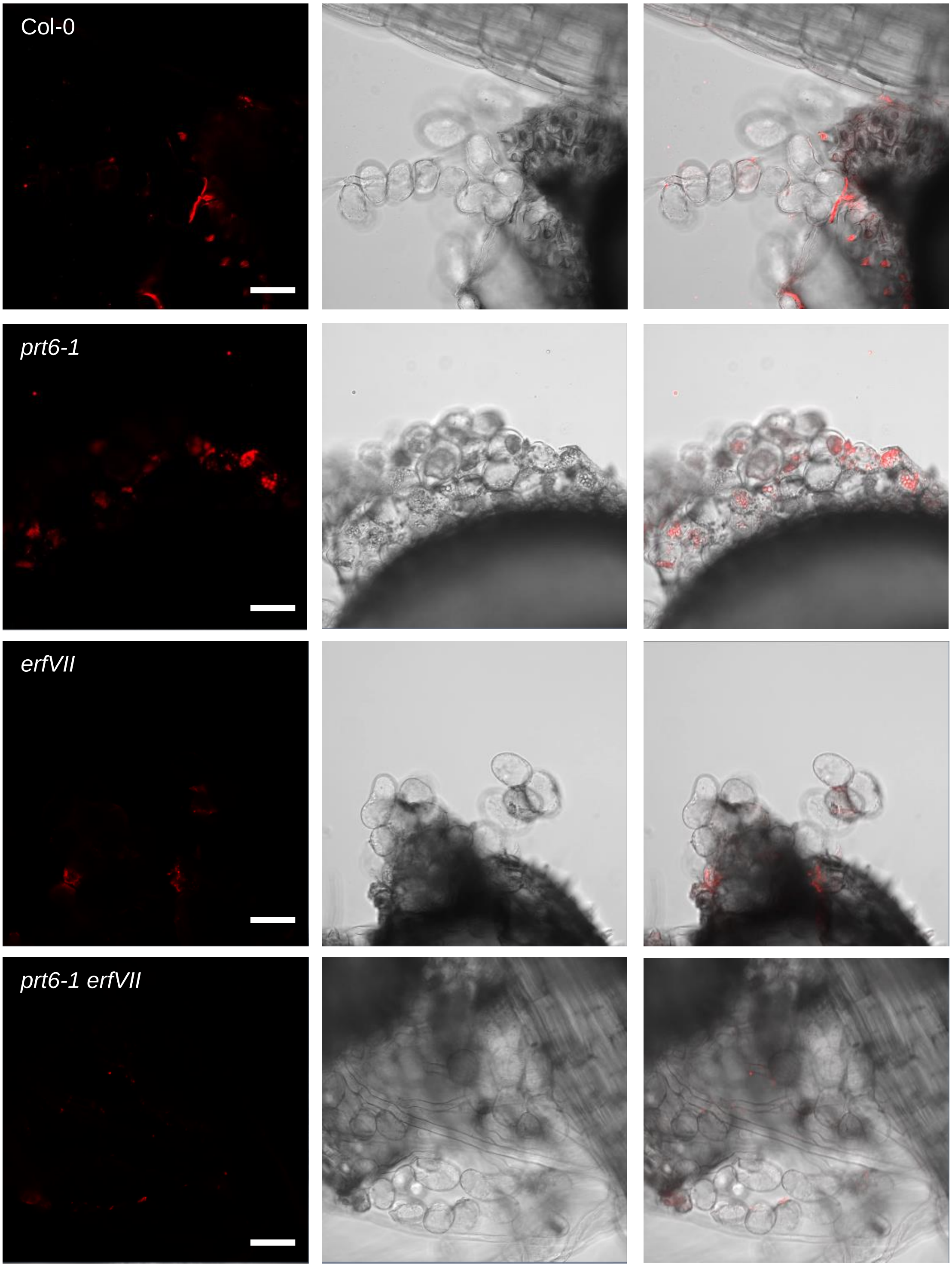

Supplementary Figure S1 Oil bodies are retained in the endosperm of *prt6* seedlings

Endosperms and seed coats were dissected from 4 d old seedlings of *prt6-1* and *erfVII* combination mutants, stained with Nile Red and visualised by confocal microscopy. Left panel: Nile Red; middle panel, bright field; right panel, merge. Bar = 50 μm.

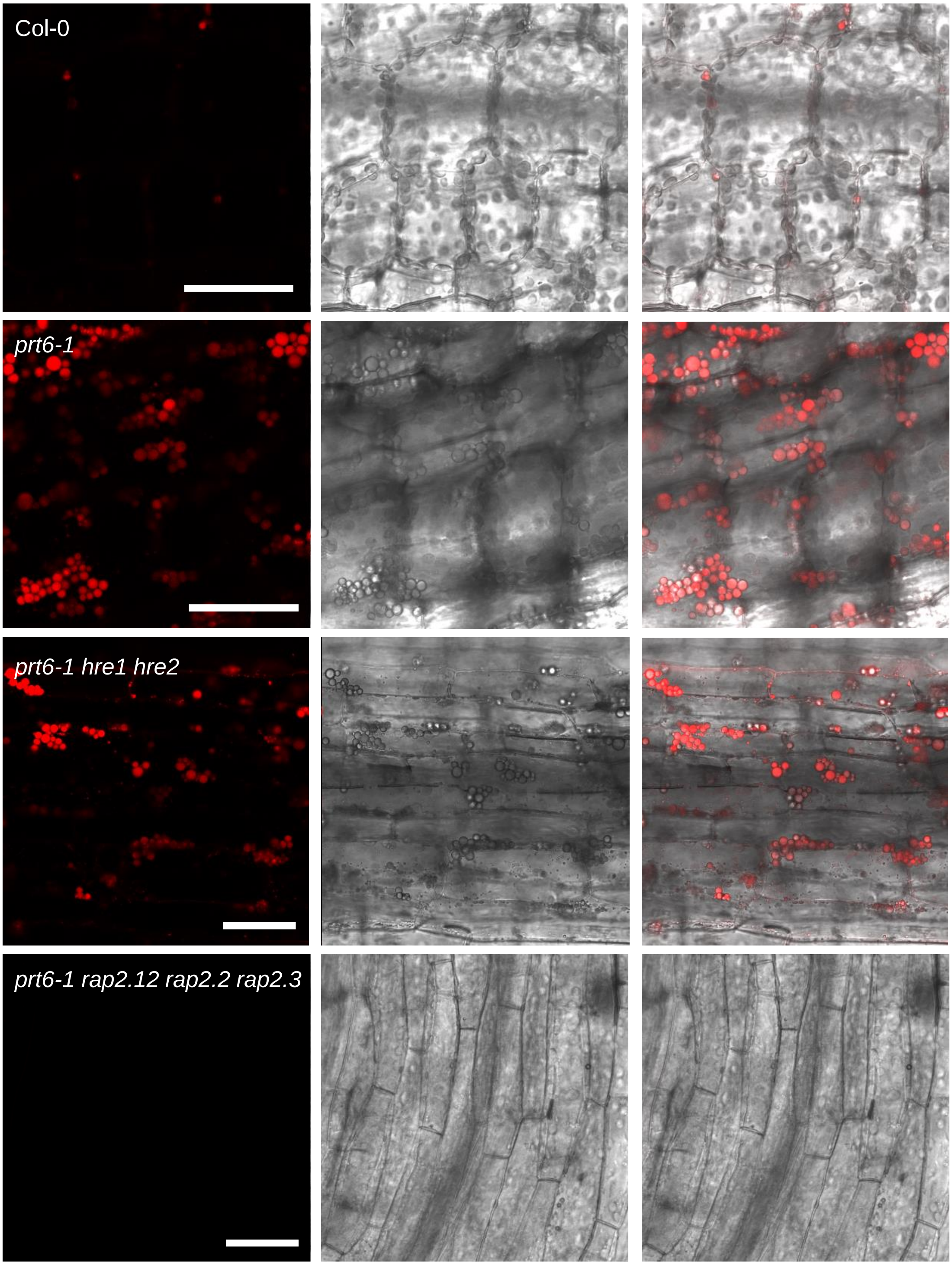

Supplementary Figure S2. Oil body retention in light-grown *prt6* seedlings is dependent on RAP-type ERFVII transcription factors
Hypocotyls of 5 d old, light-grown seedlings were stained with Nile Red and visualised by confocal microscopy. Left panel: Nile Red; middle panel, bright field, right panel, merge. Bar = 50 μm.

Figure S3

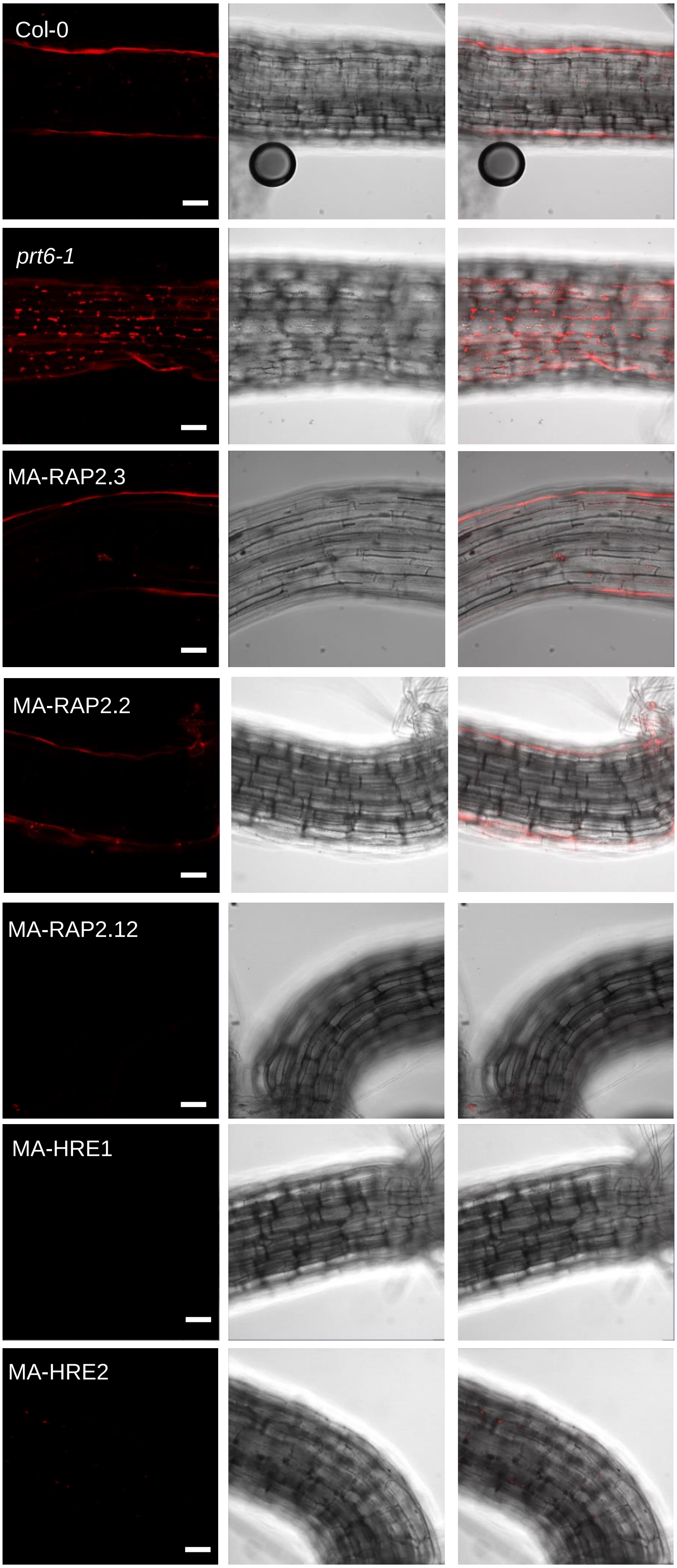

Supplementary Figure S3. Oil body phenotype of seedlings ectopically expressing stabilised ERFVII transcription factors

Hypocotyls of 5 d old, light-grown seedlings were stained with Nile Red and visualised by confocal microscopy. Results are representative of two independent transgenic lines for each construct. Left panel: Nile Red; middle panel, bright field, right panel, merge. Bar = 50 μm.

Figure S4

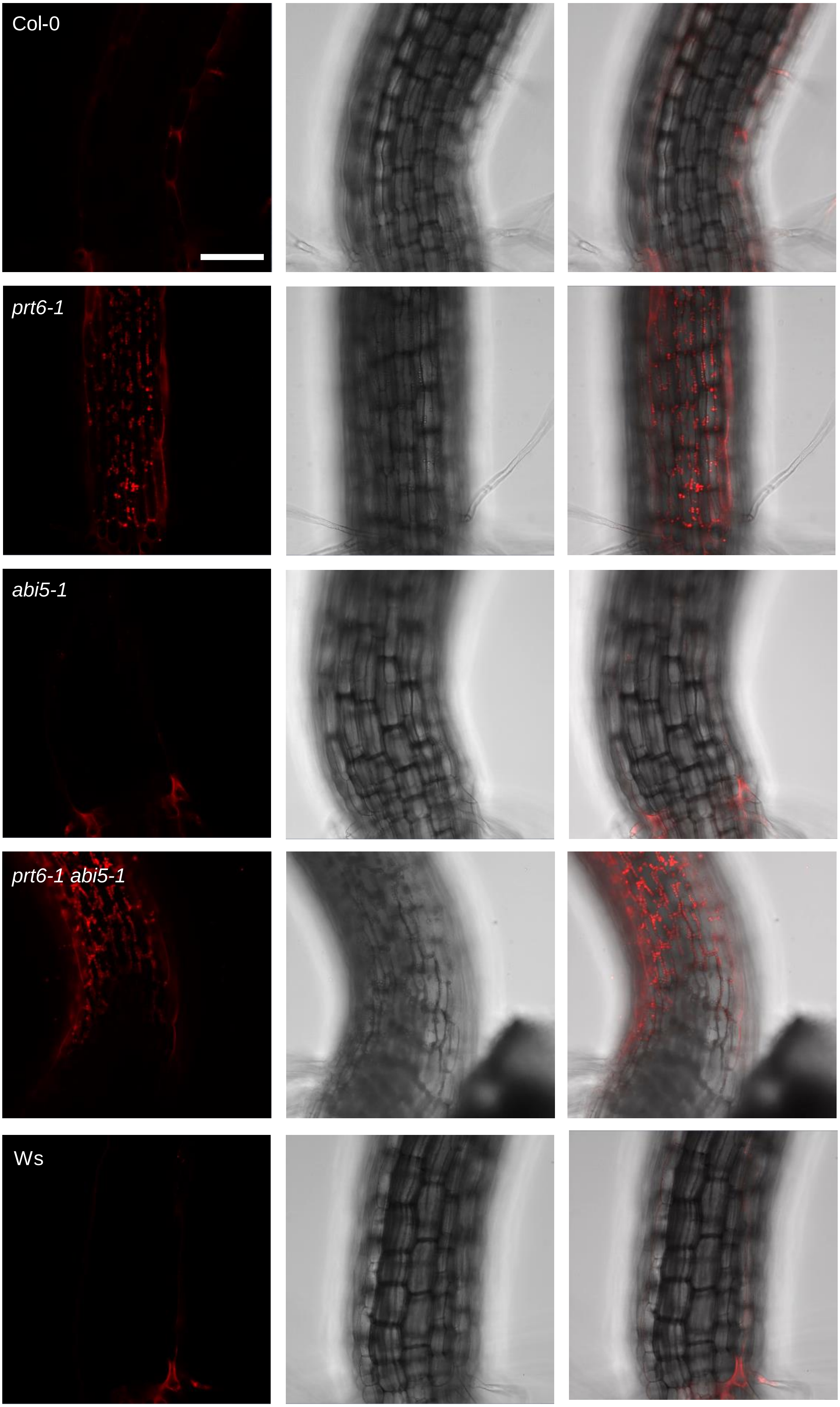

Supplementary Figure S4. Oil body phenotype of *prt6-1 abi5-1* seedlings

Hypocotyls of 5 d old, light-grown seedlings were stained with Nile Red and visualised by confocal microscopy. Left panel: Nile Red; middle panel, bright field, right panel, merge. Bar = 100 μm.

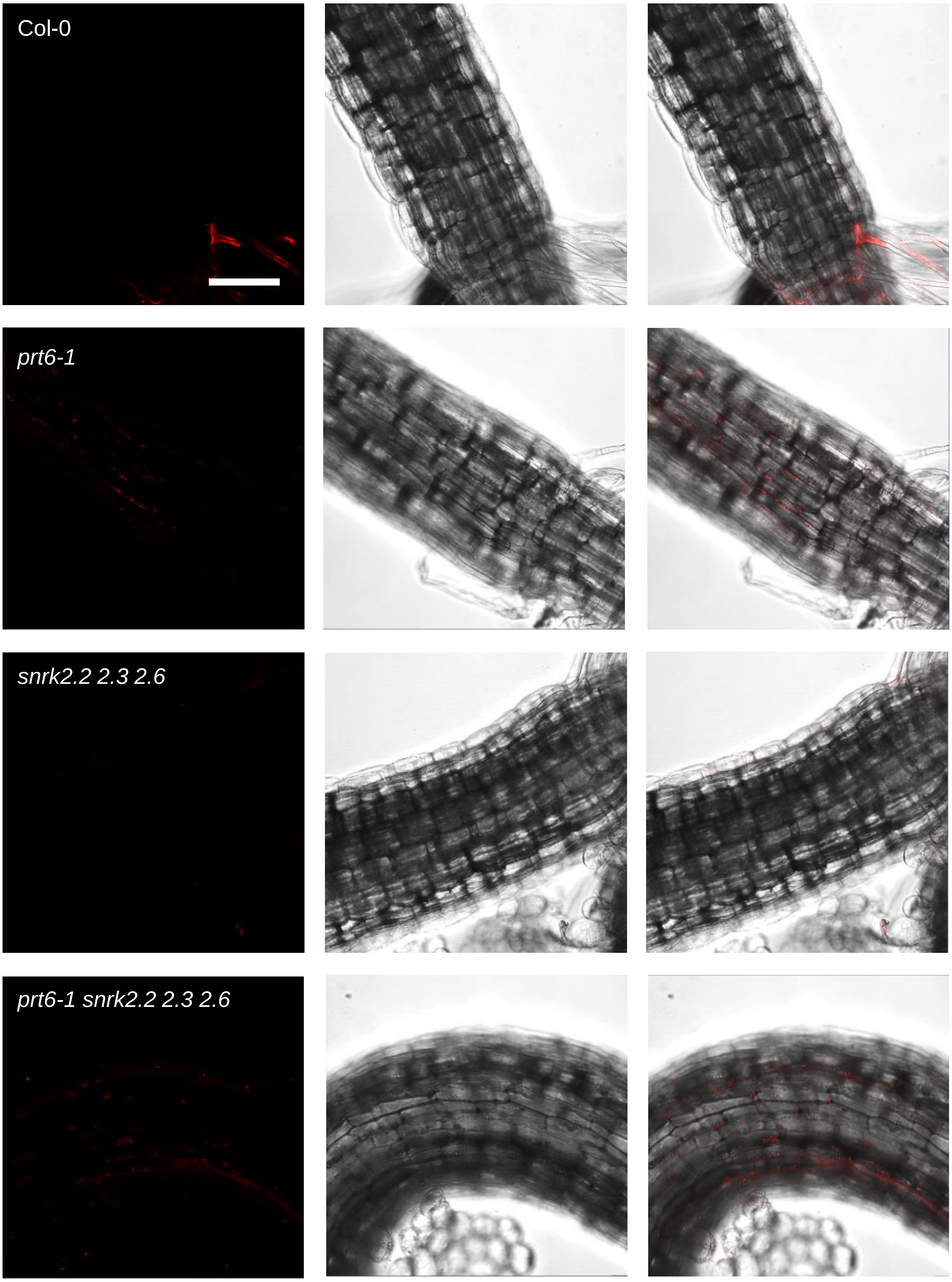

Supplementary Figure S5. Oil body phenotype of *prt6-1 snrk2.2 2.3 2.6* seedlings

Hypocotyls of 5 d old, light-grown seedlings were stained with Nile Red and visualised by confocal microscopy. Left panel: Nile Red; middle panel, bright field, right panel, merge. Bar = 100 µm.

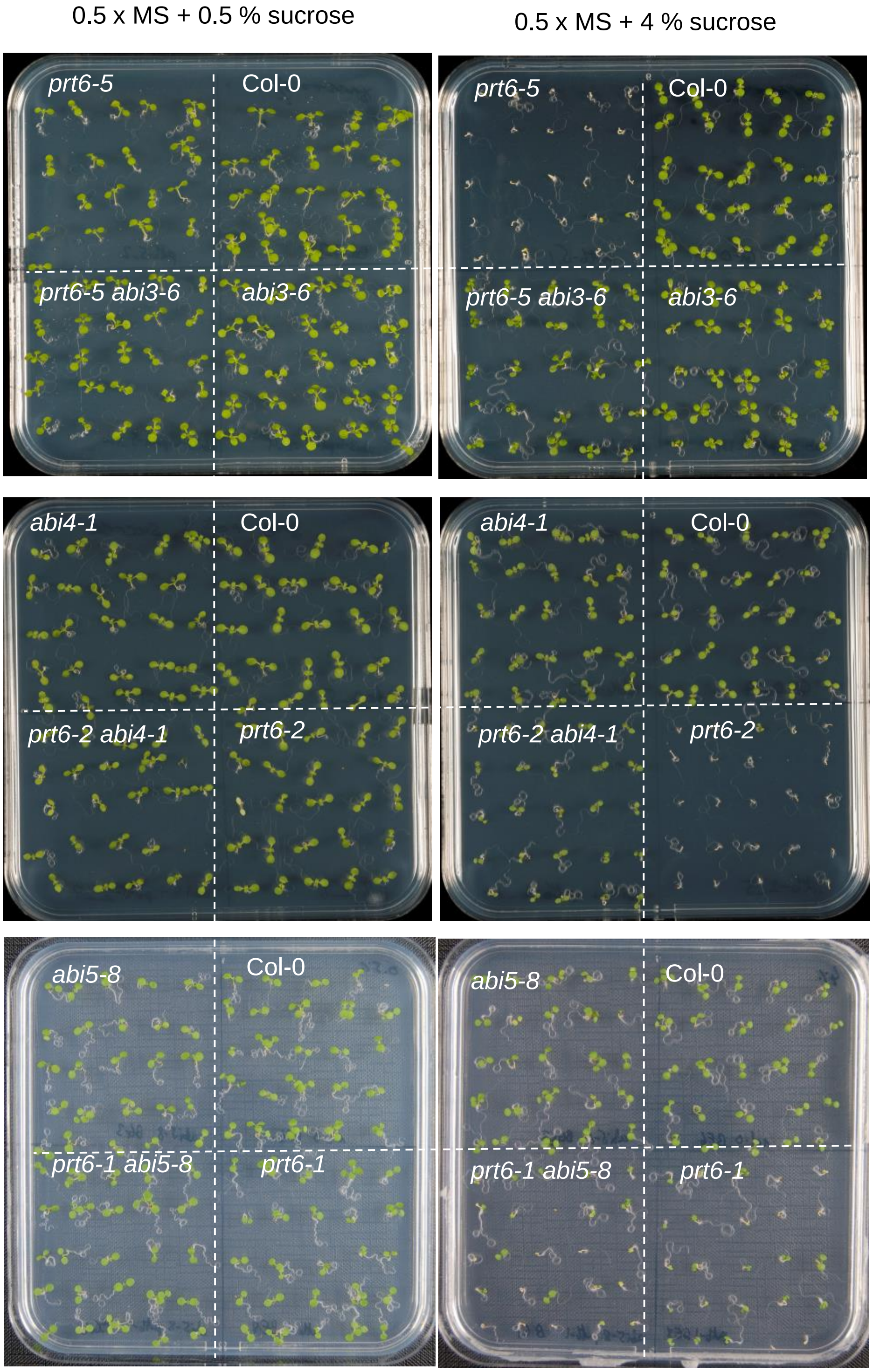

Supplementary Figure S6. Sugar sensitivity of *prt6* seedling establishment requires ABA signalling

Seeds of *prt6* and ABA signalling combination mutants were germinated on 0.5 x MS medium containing 0.5 % or 4 % sucrose. Seedlings were photographed after 5 d growth under long days.

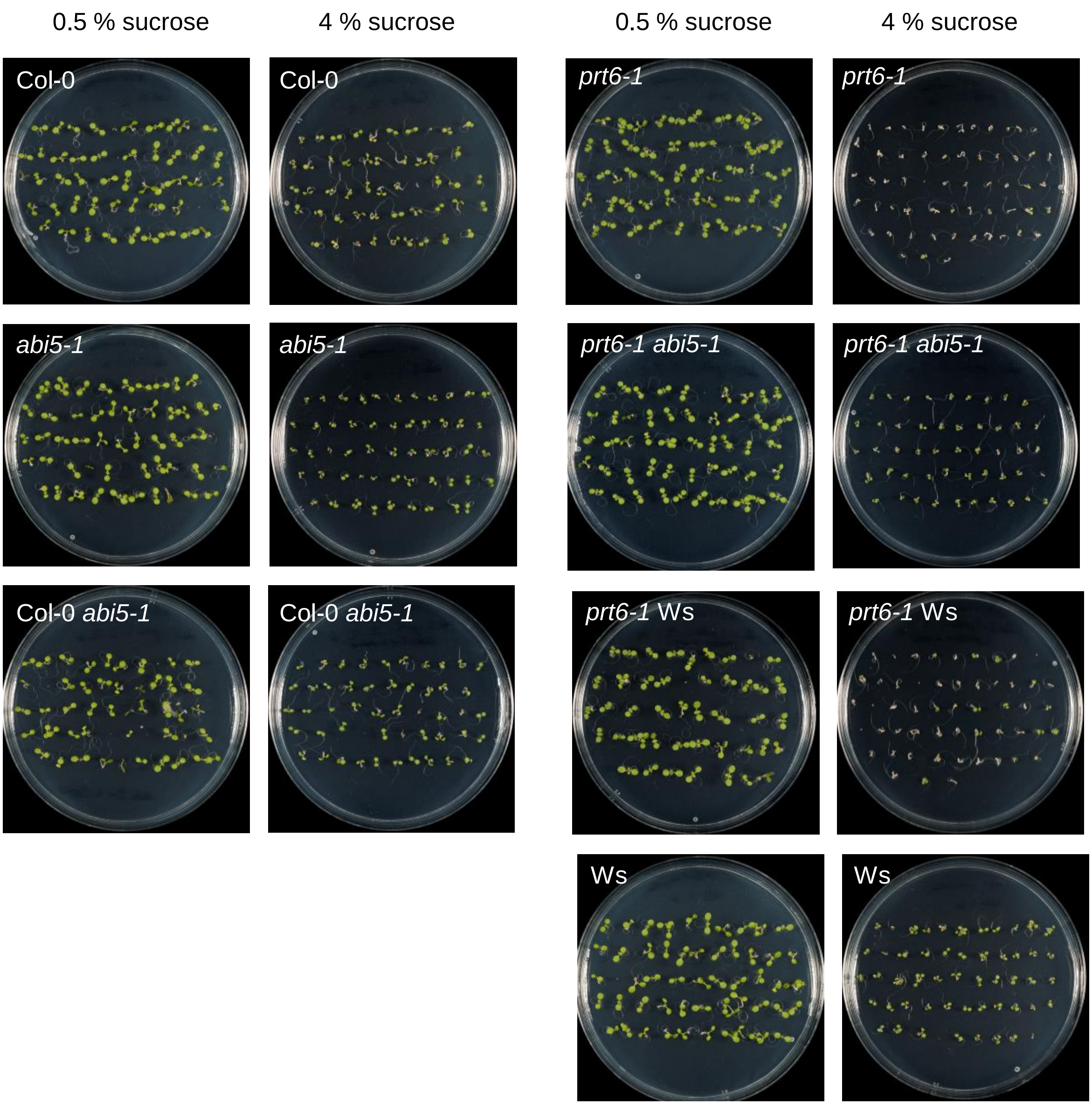

Supplementary Figure S7. Sugar sensitivity of *prt6* seedling establishment requires *ABI5*

Seeds of *prt6-1* and *abi5-1* combination mutants were germinated on 0.5 x MS medium containing 0.5 % or 4 % sucrose. Seedlings were photographed after 5 d growth under long days.

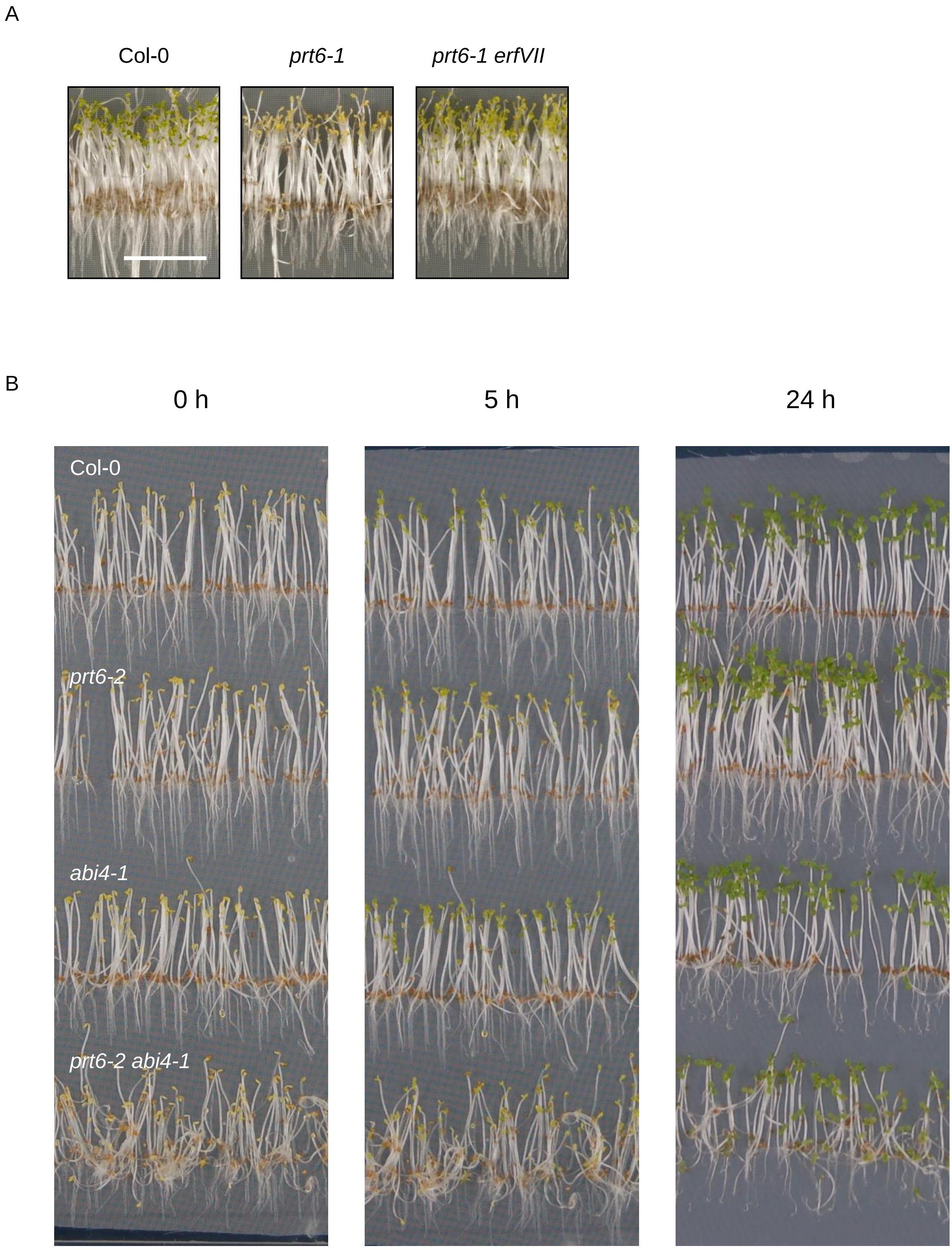

Supplementary Figure S8. Regreening of etiolated seedlings
A. Images of Col-0, *prt6-1*, and *prt6-1 erfVII* 6 h after transfer to light. Bar = 1 cm.
B. Images of Col-0, *prt6-2*, *abi4-1* and *prt6-2 abi4-1* seedlings 0, 5 and 24 h after transfer to illumination.

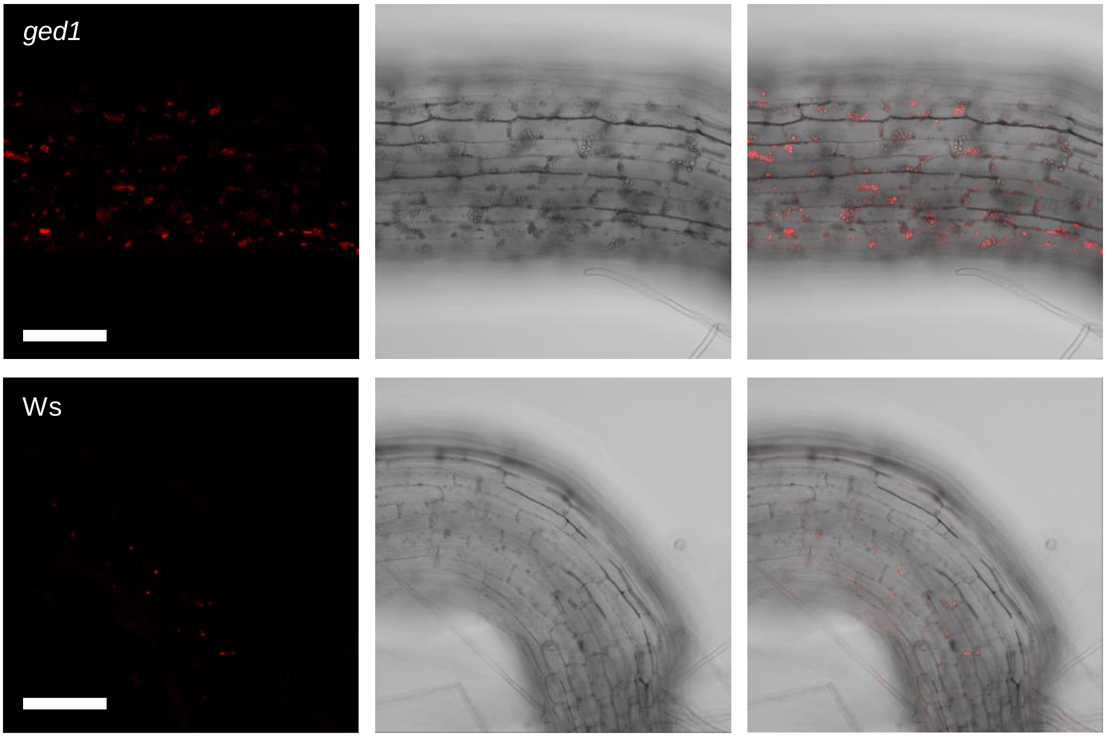

Supplementary Figure S9. Oil body phenotype of *ged1* seedlings

Hypocotyls of 5 d old, light-grown seedlings were stained with Nile Red and visualised by confocal microscopy. Left panel: Nile Red; middle panel, bright field, right panel, merge.. Bar = 100 μm

Table S1. *Arabidopsis thaliana* mutants discussed in this study

**Allele**	**Accession**	**Mutagen**	**Comments**	**Ref**
prt6-1	Col-0	T-DNA; SAIL_1278_H11	Null	19
prt6-2	Col-0	T-DNA; GK270G04	Null	19
prt6-4	L*er*	Fast neutron	Null. The *prt6-4* mutation is an insertion 497178 kb from the top of chromosome 5 into the *PRT6* (AT5G02310) gene.	19
prt6-5	Col-0	T-DNA; SALK_051088	Null	24
ged1	Ws	EMS	Null *prt6* allele. Chromosome exchange between two syntenic regions on chromosomes 3 and 5 containing 108 anchors (chromosome 3: At3g09300–At3g12320 and chromosome 5: At5g02100–At5g06980)	26
abi3-6	Col-0	Fast neutron	Null. The *abi3-6* mutation carries a 0.75 kb deletion within the region between position 1.078 and 1.944 of the *ABI3* gene. About one-third of the coding region of the *ABI3* gene is deleted.	61
abi4-1	Col-0	γ-irradiation	1 bp deletion (ΔG) in codon 157 leading to a frameshift. The resulting protein contains the presumed DNA binding and dimersiation domains but lacks the presumed activation domain and is inactive as a transcriptional activator in yeast [37].	33, 35
abi5-1	Ws	T-DNA	*abi5-1* contains a G-to-T substitution resulting in an early translation termination such that the mutant protein lacked 81 C-terminal amino acids, including the conserved basic and leucine zipper domains required for DNA binding and dimerization. The mutation does not co-segregate with the T-DNA.	36
abi5-8	Col-0	T-DNA; SALK_013163	Incomplete loss of function (less severe than *abi5-1*)	20

Table S2 Protein groups with increased abundance in the N-terminome of *prt6* etiolated seedlings that are known to be regulated by *ABI4*

Proteomics datasets from [21] were interrogated with a list of *ABI4* targets compiled from [39].

AGI code	Description
AT1G48130.1	1-cysteine peroxiredoxin 1
AT1G54870.1	NAD(P)-binding Rossmann-fold superfamily protein
AT2G28490.1	RmlC-like cupins superfamily protein
AT3G58450.1	Adenine nucleotide alpha hydrolases-like superfamily protein
AT3G15670.1	Late embryogenesis abundant protein (LEA) family protein

Table S3 Primers used in this study

**Primer**	**Sequence 5'-3'**
MA-RAP2.12_F	AAAAACGCGTATGGCTGGAGGAGCTATAATATC
RAP2.12_R	AAAGGATCCATGTGCGGAGGAGCTGTAAT
MA-RAP2.2_F	AAAAACGCGTATGGCTGGAGGAGCTATAATC
RAP2.2_R	AAATCTAGATCAAAAGTCTCCTTCCAGCAT
ABI3F	GCTGGCTCAGCTTCTGCTAT
ABI3R	AAATTCTTTTCTGGTTTCCATCC
ABI4F	CCGCTTCTTCTCCTTCCAC
ABI4R	GAGGGAGGAGAGGTCTTAGGG
ABI5F	AACATGCATTGGCGGAGT
ABI5R	TTGTGCCCTTGACTTCAAACT
ACT2F	CCGCTCTTTCTTTCCAAGC
ACT2R	CCGGTACCATTGTCACACAC
TUB4F	GCTGAGAACAGCGATTGTCTT
TUB4R	AGTTCCCATTCCAGATCCAGT
abi3-6 F3	CTTGGGCTATATTGAGATCAG
abi3-6 R6	GGCCAATACATCCAGGTCCCT
abi4-1F1	CGAGATCCGAGAGCCACGTA
abi4-1R1	GGTCCCACCATTAAGGAAGA
abi5-1 LP avaII	ACAGCTTTATGGTGTGTTTCAAGG
abi5-1 RP avaII	CCATCTGAAGACACCGGGCTTAAC
abi5-8LP3	TCACCAGCTAGAAGCTCAACG
abi5-8RP3	GCTTGGGTCATCATTATATCCG
ged1 V2 prt6 LP	TTGACATGGAACCGGTAGCC
ged1 V2 prt6 RP	TGACCATTCCCTATAAGCCGG
ged1 V2 myb3r3 LP	CGTTTGTTTCCGTTTTGTTCCG
ged1 V2 myb3r3 RP	AGTCTCAACTCCAATGACGC
